# Brain–Heart Interaction During Transcutaneous Auricular Vagus Nerve Stimulation

**DOI:** 10.3389/fnins.2021.632697

**Published:** 2021-03-15

**Authors:** Kathrin Machetanz, Levan Berelidze, Robert Guggenberger, Alireza Gharabaghi

**Affiliations:** Institute for Neuromodulation and Neurotechnology, University of Tübingen, Tübingen, Germany

**Keywords:** transcutaneous auricular vagus nerve stimulation (taVNS), non-invasive vagus nerve stimulation (nVNS), electroceutical, heart rate variability, electroencephalography, cortical oscillations

## Abstract

**Objectives:**

Transcutaneous auricular vagus nerve stimulation (taVNS) modulates brain activity and heart function. The induced parasympathetic predominance leads to an increase of heart rate variability (HRV). Knowledge on the corresponding cortical activation pattern is, however, scarce. We hypothesized taVNS-induced HRV increases to be related to modulation of cortical activity that regulates the autonomic outflow to the heart.

**Materials and Methods:**

In thirteen healthy subjects, we simultaneously recorded 64-channel electroencephalography and electrocardiography during taVNS. Two taVNS stimulation targets were investigated, i.e., the cymba conchae and inner tragus, and compared to active control stimulation in the anatomical vicinity, i.e., at the crus helicis and outer tragus. We used intermitted stimulation bursts of 25 Hz applied at a periodicity of 1 Hz. HRV was estimated with different time-domain methodologies: standard deviation of RR (SDNN), the root mean squares of successive differences (RMSSD), the percentage of RR-intervals with at least 50 ms deviation from the preceding RR-interval (pNN50), and the difference of consecutive RR intervals weighted by their mean (rrHRV).

**Results:**

The stimulation-induced HRV increases corresponded to frequency-specific oscillatory modulation of different cortical areas. All stimulation targets induced power modulations that were proportional to the HRV elevation. The most prominent changes that corresponded to HRV increases across all parameters and stimulation locations were frontal elevations in the theta-band. In the delta-band, there were frontal increases (RMSSD, pNN50, rrHRV, SDNN) and decreases (SDNN) across stimulation sites. In higher frequencies, there was a more divers activity pattern: Outer tragus/crus helicis stimulation increased oscillatory activity with the most prominent changes for the SDNN in frontal (alpha-band, beta-band) and fronto-parietal (gamma-band) areas. During inner tragus/cymba conchae stimulation the predominant pattern was a distributed power decrease, particularly in the fronto-parietal gamma-band.

**Conclusion:**

Neuro–cardiac interactions can be modulated by electrical stimulation at different auricular locations. Increased HRV during stimulation is correlated with frequency-specific increases and decreases of oscillatory activity in different brain areas. When applying specific HRV measures, cortical patterns related to parasympathetic (RMSSD, pNN50, rrHRV) and sympathetic (SDNN) modulation can be identified. Thus, cortical oscillations may be used to define stimulation locations and parameters for research and therapeutic purposes.

## Introduction

The autonomic nervous system (ANS) plays a key role in many neurological, psychiatric, cardiovascular, immunological, and metabolic disorders (Kaniusas et al., 2019). Electroceutical neuromodulation of the ANS is, therefore, investigated as a potential therapeutic intervention, when pharmacological approaches remain unsuccessful. Stimulation of the vagal nerve, which represents an essential feedback-loop between brain and body via its afferent fibers (80%) and efferent (20%), modulates the ANS toward parasympathetic predominance (Kaniusas et al., 2019). Switching points of these loops are brainstem nuclei such as the nucleus of the solitary tract (NTS, afferent), nucleus spinalis of the trigeminal nerve (NSNT, afferent), nucleus ambiguous (NA, efferent), and dorsal motor nucleus (DMN, efferent). Specifically, electrical stimulation of the external ear, referred to as transcutaneous auricular vagus nerve stimulation (taVNS), modulates a peripheral branch of the vagal nerve and is considered as a safe and well tolerated intervention, which is increasingly explored for its physiological and behavioral effects (Redgrave et al., 2018). The influence of taVNS on the feedback-loop between the ANS and the central nervous system (CNS) may be captured by different measures such as functional magnetic resonance imaging (fMRI; Kraus et al., 2013; Frangos et al., 2015; Yakunina et al., 2016; Badran et al., 2018b; Tu et al., 2018; Sclocco et al., 2019), electroencephalography (Fallgatter et al., 2003; Leutzow et al., 2013; Hagen et al., 2014), electrocardiography (Antonino et al., 2017; Badran et al., 2018c), microneurography (Clancy et al., 2014), and pupillometry (Warren et al., 2019). Multi-modal bio-signal acquisition during taVNS may provide further insights on the neurophysiological systems’ level effects, e.g., by the application of concurrent electroencephalographic and electrocardiographic recordings for the investigation of brain–heart interactions (Patron et al., 2019; Keute et al., 2021). During taVNS a shift in autonomic function toward parasympathetic predominance has been revealed by increases of heart rate variability (HRV; Clancy et al., 2014; De Couck et al., 2017; Sclocco et al., 2019); however, there are still open questions with regard to the auricular stimulation targets (Badran et al., 2018a; Burger and Verkuil, 2018), the impact on cortical brain areas and the underlying mechanisms (Leutzow et al., 2013, 2014; Polak et al., 2014) that influence the heart–brain-interaction (Silvani et al., 2016; Keute et al., 2021).

In this context, we investigated the two most often applied auricular stimulation locations, i.e., the cymba conchae and inner tragus, and the crus helicis and outer tragus as active control sites in their anatomical vicinity. We conjectured taVNS-induced HRV increases to be related to modulation of cortical activity that regulates the autonomic outflow to the heart. Since stimulation of cymba conchae and tragus has been shown to increase HRV (Clancy et al., 2014; De Couck et al., 2017), we expected differences with regard to the corresponding brain activity of these taVNS stimulation targets in comparison to the control sites that have less influence on HRV. Specifically, we conjectured these changes to occur in frontal areas which are characterized by neuro–cardiac coupling (Shoemaker et al., 2015; Ruiz Vargas et al., 2016; Patron et al., 2019).

## Materials and Methods

### Subjects

Thirteen healthy subjects (age = 24 ± 3 [mean ± SD], 8 female) took part in this exploratory proof-of-concept study, which was a secondary data analysis of previous work on the impact of different auricular stimulation locations and parameters on HRV (Machetanz et al., under review). The electroencephalography data that we analyzed here has not been reported before. The experimental procedure and study-related information is identical to the previous report and is cited here accordingly when applicable (Machetanz et al., under review): Healthy, right-handed adults aged 18–80 years were included as participants. Exclusion criteria were checked beforehand and comprised an Edinburgh Handedness Inventory score below 75, any history of habitual drug or alcohol consumption, pregnancy, cardiac diseases, cognitive, or psychiatric impairments or neurological disorders. Furthermore, patients were asked to avoid substances that alter autonomic activity (e.g., caffeine or alcohol on the day of measurement for at least 2 h before the examination). Subjects gave their written informed consent before participation. The study was approved by the local ethics committee of the medical faculty of the University of Tübingen.

### Experimental Procedure

**FIGURE 1:**
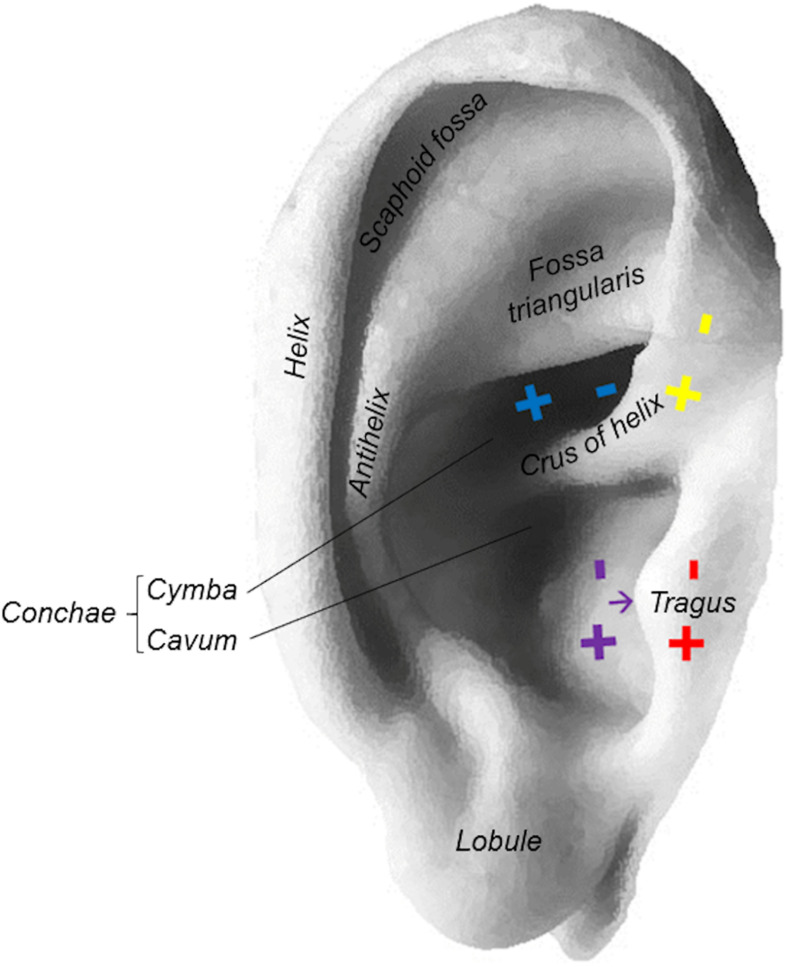
Stimulation locations at the ear: cymba conchae (blue), inner tragus (purple), outer tragus (red), crus helicis (yellow).

Within one stimulation session, participants received taVNS to six different stimulation locations at either the right (*n* = 7; age = 24.3 ± 2.8; 3 females) or left ear (*n* = 6; age = 24 ± 3.1; 5 females). The stimulation locations were determined due to anatomical landmarks (e.g., separating the concha into cymba and cavum according to the crus of helix; Figure 1). There was no blinding of the participations or the examiner regarding the side of stimulation. However, subjects were not informed about the different stimulation localizations. Due to the low sample size, participants with right and left ear stimulation were pooled for the purpose of the present study.

At each stimulation location and parameter combination, we applied 30 bursts with a periodicity of 1 Hz, while each burst consisted of five pulses applied at 25 Hz. We started with the lowest electrical charge in three different charge-balanced parameter combinations [i.e., 100 μs (0.250 mA), 260 μs (0.096 mA), and 500 μs (0.050 mA)]. This resulted in 90 s (i.e., 3 × 30 s) of stimulation at the same charge and stimulation location. Then, the next higher charge was applied [i.e., 100 μs (0.500 mA), 260 μs (0.192 mA), and 500 μs (0.100 mA)]. After applying 2–3 different charges at one location, there was a short break of around 1 min before moving to the next location. Subsequently, the next stimulation round with higher charges ensued. Thereby, eight different electrical charges were evaluated by investigating three pulse durations and eight charge-balanced current intensities, i.e., 100 μs (0.250–2 mA in steps of 0.250 mA), 260 μs (0.096–0.769 mA in steps of 0.096 mA), and 500 μs (0.050–0.400 mA in steps of 0.050 mA).

### Data Acquisition

**FIGURE 2:**
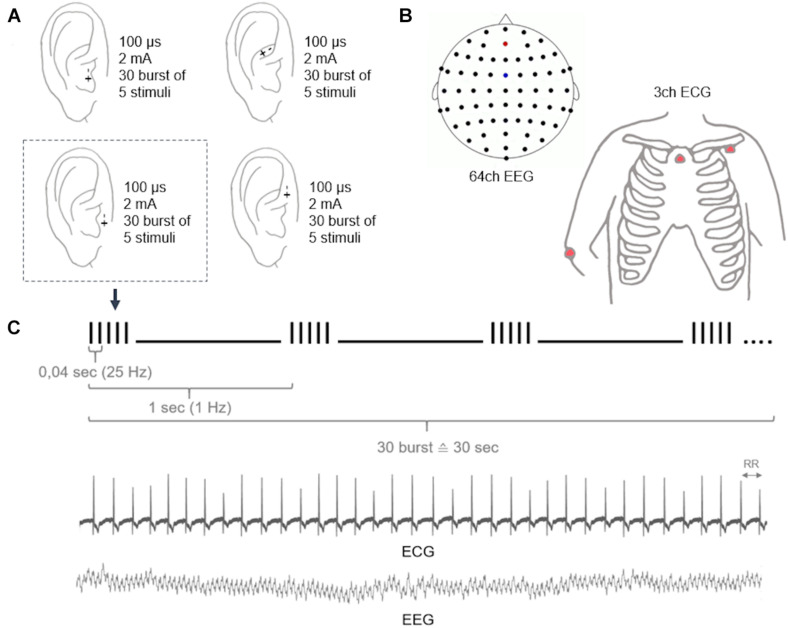
Overview of the stimulation procedure during concurrent ECG/EEG recordings.

The participants were positioned in a comfortable reclining chair, and electrocardiography (ECG), electroencephalography (EEG), and electrooculography (EOG) were recorded simultaneously. All signals were digitalized at a sampling frequency of 1,000 Hz (Brain Products Amplifiers, Brain Products GmbH, Gilching, Germany). Here, we analyzed the relationship between HRV obtained from a 3-channel ECG (electrodes at the left clavicle, sternum, and right olecranon) and EEG power spectrum from 64 Ag/AgCl electrodes (reference: FCz, ground: AFz) in accordance with the international 10–20 system (Figure 2). For artifact rejection, EOG was determined by Ag/AgCl electrodes above and below the left eye as well as a reference at the right olecranon.

For stimulation, we used a multichannel stimulator (STG-4000 series, multichannel systems, Harvard Bioscience Inc.) that was triggered by a customized python program and which allows current as well as voltage driven stimulation. Furthermore, a handheld bipolar spherical probe (GVB-gelimed GmbH) with a probe diameter of 2 mm and an inter-probe distance of 5 mm was applied. Throughout the experiment, participants were at rest; they were asked to relax and to indicate uncomfortable sensations whenever they occurred during stimulation. Stimulation was terminated, if the sensations became uncomfortable for the subjects. However, the stimulation intensity was not adjusted according to the individual perception threshold as applied in other studies (Clancy et al., 2014; Badran et al., 2018c; Borges et al., 2020), because (i) such adjustments were used inconsistently in previous studies and (ii) our aim was to stimulate the Aß-fibers and not the Aδ- and C-fibers, which primarily convey pain stimuli.

### Data Analysis and Statistics

The ECG analysis of our previous study revealed that taVNS of the cymba conchae, and to a lesser extend also of the inner tragus, had the strongest influence on HRV increases (Machetanz et al., under review). Therefore, we chose the stimulation at these two locations as the verum intervention for our present EEG analysis. More specifically, we investigated the cortical oscillatory activity corresponding to HRV increases during taVNS of the cymba conchae and the inner tragus. For our proof-of-concept study, we restricted our analysis to stimulation with one parameter combination. Specifically, we investigated the cortical effects of stimulation with an amplitude of 2 mA and a pulse width of 100 μs, since our previous study revealed that this parameter combination was both effective in increasing HRV and comfortable for the participants. Moreover, a pulse width of 100 μs allows for a more selective fiber recruitment than longer pulse durations (Gorman and Mortimer, 1983; Helmers et al., 2012). Furthermore, a current intensity of 2 mA (which is lower than in other studies) allows for the activation of a restricted auricular region, i.e., the verum and the active sham condition can be applied in close vicinity without overlap of the stimulation areas (Figure 1). This allowed us to select stimulation of the crus helicis and outer tragus as active control conditions. More specifically, these stimulation locations are in the anatomical vicinity of the cymba conchae and inner tragus, respectively, but were shown to modulate the HRV significantly less (Machetanz et al., under review). We intentionally did not choose the common control site, i.e., the ear lobe, due to its distance to the cymba conchae and inner tragus. Thereby, we intended to avoid that the different density of perivascular sympathetic neurotransmitters that varies between lower and upper auricular zones would bias the stimulation effects (Cakmak, 2019).

The analysis of electrophysiological data was performed using MATLAB (MathWorks, Inc., Natick, MA, United States), Fieldtrip open-source toolbox^1^ and other compatible toolboxes. EEG pre-processing included an automated artifact rejection by a linear regression of EOG data, a visual artifact rejection and epoching of the continuous EEG data into segments of 500 ms (350–850 ms after the first pulse). In a subsequent time-frequency analysis, the power spectrum of delta (0.5–3.5 Hz), theta (4–7 Hz), alpha (8–14 Hz), beta (15–30 Hz), and gamma (31–45 Hz) oscillatory activity was analyzed separately for each of the aforementioned segments using the methodology of a discrete prolate spheroidal sequence (DPSS; Percival and Walden, 1993).

For the ECG analysis, we used the MarcusVollmer/HRV toolbox.^2^ The automated artifact rejection and R-peak detection was visually inspected to identify and correct for false positive and false negative findings. Subsequently, we applied different time-domain methodologies for HRV estimation by means of the toolbox, i.e., the standard deviation of RR (SDNN), the root mean squares of successive differences (RMSSD), and the pNN50 defined as the percentage of RR-intervals with at least 50 ms deviation from the preceding RR-interval. The SDNN is regarded as a parameter for the overall HRV (i.e., sympathetic and parasympathetic), while pNN50 and RMSSD are considered to reflect the parasympathetic nervous system. In addition, we analyzed the rrHRV which is based on the difference of consecutive RR intervals weighted by their mean, and which is particularly applicable for short RR sequences (Vollmer, 2015).

The study protocol followed a specific order of the applied parameters (see section “Experimental procedure”). Therefore, we controlled for a possible order effect of the performed examination runs by defining a general linear model with the run of each block as a categorical factor. Specifically, we regressed it out of the various HRV parameters and the 64 channels of the EEG power spectrum. Subsequently, we performed a correlation analysis for each EEG channel (of each power spectrum) with each HRV parameter (normalized for order) for the selected parameter conditions (2 mA, 100 μs; stimulation at cymba conchae/inner tragus and outer tragus/crus helicis), and determined the correlation matrix on the basis of pairwise Pearson correlations. Finally, the correlation coefficient (*R*-value) and the significance level (*P*-value) were graphically depicted in color-coded topoplots with a blue-to-yellow scale or black-to-white scale, respectively. The analyses were performed for the pooled trials of the inner tragus and cymbae conchae vs. the pooled trials of the crus helicis and outer tragus, and after concatenating left and right ear stimulation, to increase the number of trials for each condition (verum vs. active control).

## Results

**FIGURE 3:**
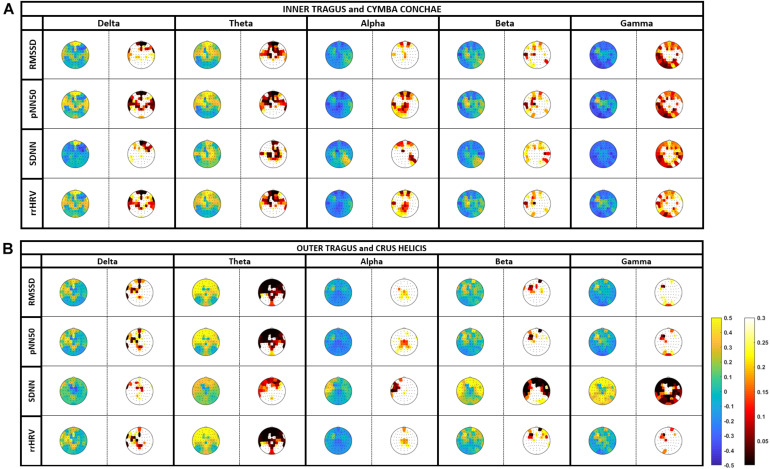
Correlation between heart-rate variability (HRV) and cortical oscillatory activity (EEG): tVNS was applied at the cymba conchae/inner tragus and outer tragus/crus helicis with 2 mA and 100 μs. Oscillatory activity is estimated in the delta (0.5–3.5 Hz), theta (4–7 Hz), alpha (8–14 Hz), beta (15–30 Hz), and gamma (31–45 Hz). Color-coded topoplots show *r*-values (blue-to-yellow scale) and *p*-values (black-to-white scale) of the correlation analysis, where blue and black indicate power reduction and significance, respectively.

The stimulation-induced HRV increases corresponded to frequency-specific oscillatory modulation of different cortical areas. All stimulation targets induced power modulations that were proportional to the HRV elevation (Figure 3 and Supplementary Tables 1, 2).

The most prominent changes that corresponded to HRV increases across all measures (RMSSD, pNN50, rrHRV, and SDNN) and stimulation locations (cymba conchae/inner tragus, crus helicis/outer tragus) were significant frontal elevations (region of interest, ROI: Fp1, Fp2, AF3, AF4, Fpz, Fz) in the oscillatory theta-band. The mean *P*-value of a frontal region of interest (ROI; Fp1, Fp2, AF3, AF4, Fpz, Fz) was significant for RMSSD (*P*-value: 0.05 ± 0.06), pNN50 (0.04 ± 0.05) and rrHRV (0.04 ± 0.23) during cymba conchae/inner tragus stimulation; and also during crus helicis/outer tragus stimulation (*P*-value: <0.001 ± 0.001; <0.001 ± 0.0004; 0.001 ± 0.0005, respectively).

In the delta-band, there were frontal increases (RMSSD, pNN50, rrHRV, SDNN) and decreases (SDNN) across stimulation sites. However, these changes did not reach significance. In higher frequency bands, there was a more divers activity pattern: Outer tragus/crus helicis stimulation increased oscillatory activity with the most prominent changes for the SDNN measure in frontal (alpha-band, beta-band) and fronto-parietal (gamma-band) areas, which reached significance in the frontal ROI beta-band (*P*-value: 0.004 ± 0.01) and gamma-band (*P*-value: <0.001 ± 0.0004). Inner tragus/cymba conchae stimulation led also to circumscribed alpha- and beta-band increases; however, the predominant pattern was a distributed power decrease, particularly in the fronto-parietal gamma-band.

## Discussion

Despite its wide range of potential therapeutic applications, there still exists controversy with regard to the underpinnings of taVNS. While the vagal influence on the ANS is established, the underlying anatomical and physiological mechanisms of the vagal CNS–ANS feedback loop are still incompletely understood (Cakmak, 2019; Kaniusas et al., 2019). In this context, the aim of this study was to evaluate corresponding changes of HRV and cortical activation patterns during taVNS. The present work showed, that the increase of HRV correlated with specific cortical activation patterns in different frequency bands.

Our results indicated a frontal increase in delta/theta oscillatory activity to be associated with an HRV increase during inner tragus and cymba conchae stimulation. This is in line with previous studies that revealed a neuro–cardiac coupling between HRV parameters and delta band activity in the frontal cortex as a cortical area promoting cardiac parasympathetic activity (Thayer et al., 2012; Patron et al., 2019; Keute et al., 2021). These studies showed higher cardiac control during stronger neuro–cardiac coupling. Interestingly, EEG correlates of 10 Hz vagus nerve stimulation for Crohn’s disease showed acute increases of delta and theta activity as well (Kibleur et al., 2018). This observation is also supported by previous fMRI studies, which observed an association between increased vagal cardiac control and elevated activity in frontal areas (Shoemaker et al., 2015; Ruiz Vargas et al., 2016).

However, our data showed a frontal increase in delta/theta oscillatory activity also during active control stimulation at the outer tragus and crus helicis. This may be interpreted in different ways: The increase in delta/theta activity (that correlated with HRV) may not reflect specific vagal effects, but processes related to the general sensory afference induced by peripheral electrical stimulation (Hansen and Nielsen, 2004) or to changes of the attentional state (Aftanas and Golocheikine, 2001). Thereby, these findings may support the differentiation of autonomic, sensory and cognitive effects of auricular stimulation in future work. An alternative interpretation may suggest that stimulation at the active control locations was activating branches of the auricular branch of the vagus nerve – or other mechanisms that influence HRV – as well. Specifically, due to the proximity of the crus helicis and outer tragus to the cymba conchae and the inner tragus, respectively, electrical current may have been disseminated to more distantly located vagal fibers despite the relatively low stimulation intensity and narrow pulse width applied. An anatomical study of the auricular nerve supply in human cadavers revealed the most consistent vagal innervation patterns for the cymba conchae (Peuker and Filler, 2002), whereas, e.g., the crus helicis and fossa triangularis were innervated also by the lesser occipital nerve (LON), greater auricular nerve (GAN), and the auricotemporal nerve (ATN). However, although the outer tragus has been used as a location for taVNS in previous work (Badran et al., 2018b), the innervation of the tragus remains unclear due to inconsistent reporting (Peuker and Filler, 2002; Badran et al., 2018a; Burger and Verkuil, 2018). HRV-changes during cymba conchae and tragus stimulation (which are the two most investigated auricular stimulation targets) may be elicited by different mechanisms as stated elsewhere (Machetanz et al., under review): A fMRI study showed activation of vagal brainstem nuclei during stimulation of both targets, but with significant increases in comparison to control stimulation at the ear lobe for the cymba conchae only (Yakunina et al., 2016). On the other side, EEG recordings showed cortical evoked potentials, referred to as vagus somatosensory evoked potentials (VSEP), during inner tragus but not cymba conchae stimulation (Fallgatter et al., 2003). Notably, these EEG responses disappeared under a pharmacological neuromuscular block (Leutzow et al., 2013), which suggests rather a peripheral muscular than a central neural origin and has, therefore, initiated a debate with regard to the underlying mechanisms (Leutzow et al., 2014; Polak et al., 2014). In this context, two recent studies in humans (Clancy et al., 2014) and rats (Mahadi et al., 2019) suggested an alternative mechanisms of the observed HRV effects during tragus stimulation: microneurography recordings revealed a decrease of muscle sympathetic nerve activity (Clancy et al., 2014); recordings from the spinal sympathetic chain showed also an inhibition of sympathetic nerve activity, which disappeared after cervical nerve transections (Mahadi et al., 2019). These findings suggested that tragus stimulation effected HRV by potentially different autonomic mechanisms and pathways than cymba conchae stimulation. Furthermore, the influence of stimulation of auricular muscle zones, perivascular sympathetic neurotransmitters and the localization of perforatory artery zones should not be underestimated (Cakmak, 2019). Therefore, the similar activation pattern in frontal areas during stimulation of the classical tVNS targets and active control localizations could be induced by different mechanisms. Further studies with larger cohorts are necessary to better understand the observations.

In higher frequency bands, stimulation at tVNS targets (inner tragus/cymba conchae) and control localizations (outer tragus/crus helicis) showed a more divers and – in part – opposing pattern: Outer tragus/crus helicis stimulation increased oscillatory activity with the most prominent cortical changes correlating with HRV for the SDNN measure in frontal (alpha-, beta-band) and fronto-parietal (gamma-band) areas. Inner tragus/cymba conchae stimulation led also to circumscribed alpha- and beta-band increases; however, the predominant pattern was a distributed power decrease, particularly in the fronto-parietal gamma-band that correlated with HRV.

Notably, modulation of these higher frequency bands can be observed during somatosensory activation/processing as well as various cognitive functions (i.e., attention and memory; Herrmann et al., 2004; Jensen et al., 2007). Accordingly, in a case of Crohn’s disease, vagus nerve stimulation led to negative and positive correlation between HRV and gamma-band activity in frontal and parieto-occipital areas, respectively (Clarençon et al., 2014). In this context, previous studies showed also a divers and even contradictory picture. In tinnitus and epilepsy patients, for example, the previously increased gamma activity was reduced by vagus nerve stimulation (Willoughby et al., 2003; Hyvarinen et al., 2015); whereas, another study with epilepsy patients revealed a VNS-induced increase in gamma activity (Marrosu et al., 2005). These findings were interpreted as seizure-independent modulations of attention networks. Thus, the different effects of cymba conchae/inner tragus and outer tragus/crus helicis stimulation on gamma-band activity in the present study may reflect a different modulation of attention and/or the sympathetic system that is captured by the SDNN measure as well.

### Limitations

This proof-of-concept study investigated EEG/ECG periods of 30 s only, since it was a secondary analysis of a screening session with different stimulation locations and parameters. Although even (ultra) short ECG recordings have been shown to be valid for HRV estimation (Percival and Walden, 1993; Kilner et al., 2005; Munoz et al., 2015; Shaffer and Ginsberg, 2017) and concurrent EEG/ECG evaluation (Patron et al., 2019), future studies may apply longer measurement periods including additional HRV parameters such as the LF/HF ration. Furthermore, on the basis of the present work, the long-tern effects of the novel intermitted taVNS paradigm on brain–heart interactions may be compared to standard continuous stimulation protocols. Moreover, future work would need to also consider trigeminal and sympathetic circuits by additional measures such as electromyography, microneurography, and pupillometry, and investigate larger sample sizes to allow for more detailed comparisons, e.g., between right vs. left ear stimulation.

## Conclusion

Increased HRV during auricular stimulation is correlated with frequency-specific increases and decreases of oscillatory activity in different brain areas.

The HRV measures RMSSD, pNN50, and rrHRV, reflecting the parasympathetic nervous system, revealed consistent findings across frequency bands. With these measures, the most prominent changes that corresponded to HRV increases were frontal elevations in the oscillatory theta-band; they occurred across stimulation locations, i.e., during stimulation of cymba conchae/inner tragus and crus helicis/outer tragus.

The HRV measure SDNN, reflecting both the parasympathetic and sympathetic nervous system, differed from the other measures; the most prominent changes that corresponded to HRV were increased oscillatory activity in frontal (alpha-band, beta-band) and fronto-parietal (gamma-band) areas during outer tragus/crus helicis stimulation; while inner tragus/cymba conchae stimulation led predominantly to a distributed power decrease, particularly in the fronto-parietal gamma-band.

In summary, cortical oscillations may be used to define stimulation locations and parameters for research and therapeutic purposes.

## Data Availability Statement

The raw data of the presented study can be provided on request.

## Ethics Statement

The studies involving human participants were reviewed and approved by Ethics Committee of the Medical Faculty of the University of Tübingen. The patients/participants provided their written informed consent to participate in this study.

## Author Contributions

KM contributed to the conception and design of the study, the acquisition, analysis and interpretation of data, and writing of the manuscript. LB and RG contributed to the data acquisition, analysis, interpretation of data, and review of the manuscript. AG was responsible for the conception and design of the study, interpretation of data, and writing of the manuscript. All authors contributed to the article and approved the submitted version.

## Conflict of Interest

The authors declare that the research was conducted in the absence of any commercial or financial relationships that could be construed as a potential conflict of interest.

## Footnotes

1 http://fieldtrip.fcdonders.nl/

2 https://marcusvollmer.github.io/HRV (v0.98; 02.09.2019).
