# Supplementary material for: Brain–Heart Interaction During Transcutaneous Auricular Vagus Nerve Stimulation
Source: Front Neurosci. 2021 Mar 15;15:632697. doi: 10.3389/fnins.2021.632697 (PMC8005577; doi:10.3389/fnins.2021.632697)
Supplement: Supplementary file 1 [file Table_1.docx]

|  | **DELTA** | | | | **THETA** | | | | **ALPHA** | | | | **BETA** | | | | **GAMMA** | | | |
| --- | --- | --- | --- | --- | --- | --- | --- | --- | --- | --- | --- | --- | --- | --- | --- | --- | --- | --- | --- | --- |
|  | **RMSSD** | **pNN50** | **SDNN** | **rrHRV** | **RMSSD** | **pNN50** | **SDNN** | **rrHRV** | **RMSSD** | **pNN50** | **SDNN** | **rrHRV** | **RMSSD** | **pNN50** | **SDNN** | **rrHRV** | **RMSSD** | **pNN50** | **SDNN** | **rrHRV** |
| **Fp1** | 0,049 | 0,027 | 0,621 | 0,042 | 0,035 | 0,027 | 0,512 | 0,041 | 0,105 | 0,041 | 0,114 | 0,057 | 0,218 | 0,198 | 0,299 | 0,182 | 0,148 | 0,153 | 0,168 | 0,156 |
| **Fp2** | 0,001 | 0,011 | 0,029 | 0,007 | 0,003 | 0,005 | 0,050 | 0,005 | 0,082 | 0,039 | 0,116 | 0,053 | 0,157 | 0,136 | 0,195 | 0,194 | 0,123 | 0,108 | 0,163 | 0,154 |
| **F3** | 0,699 | 0,353 | 0,452 | 0,359 | 0,052 | 0,021 | 0,373 | 0,015 | 0,571 | 0,652 | 0,873 | 0,517 | 0,205 | 0,104 | 0,511 | 0,110 | 0,502 | 0,306 | 0,744 | 0,269 |
| **F4** | 0,103 | 0,248 | 0,058 | 0,234 | 0,028 | 0,077 | 0,027 | 0,066 | 0,349 | 0,140 | 0,457 | 0,189 | 0,213 | 0,200 | 0,190 | 0,256 | 0,163 | 0,138 | 0,195 | 0,213 |
| **C3** | 0,290 | 0,068 | 0,949 | 0,155 | 0,021 | 0,038 | 0,025 | 0,045 | 0,880 | 0,527 | 0,335 | 0,468 | 0,154 | 0,083 | 0,612 | 0,182 | 0,361 | 0,618 | 0,135 | 0,525 |
| **C4** | 0,518 | 0,131 | 0,453 | 0,222 | 0,056 | 0,201 | 0,010 | 0,172 | 0,624 | 0,927 | 0,167 | 0,851 | 0,418 | 0,306 | 0,900 | 0,483 | 0,405 | 0,514 | 0,166 | 0,581 |
| **P3** | 0,905 | 0,726 | 0,854 | 0,655 | 0,519 | 0,688 | 0,204 | 0,600 | 0,401 | 0,126 | 0,895 | 0,167 | 0,925 | 0,969 | 0,924 | 0,996 | 0,119 | 0,147 | 0,267 | 0,325 |
| **P4** | 0,687 | 0,851 | 0,938 | 0,887 | 0,592 | 0,787 | 0,146 | 0,708 | 0,908 | 0,404 | 0,133 | 0,389 | 0,806 | 0,906 | 0,385 | 0,757 | 0,057 | 0,026 | 0,198 | 0,093 |
| **O1** | 0,972 | 0,607 | 0,966 | 0,776 | 0,825 | 0,745 | 0,559 | 0,794 | 0,394 | 0,212 | 0,954 | 0,217 | 0,233 | 0,175 | 0,487 | 0,188 | 0,033 | 0,025 | 0,099 | 0,050 |
| **O2** | 0,913 | 0,763 | 0,974 | 0,820 | 0,938 | 0,827 | 0,387 | 0,886 | 0,741 | 0,462 | 0,875 | 0,456 | 0,474 | 0,792 | 0,285 | 0,636 | 0,224 | 0,204 | 0,291 | 0,411 |
| **F7** | 0,380 | 0,104 | 0,518 | 0,229 | 0,310 | 0,086 | 0,646 | 0,172 | 0,691 | 0,875 | 0,202 | 0,794 | 0,217 | 0,283 | 0,194 | 0,236 | 0,084 | 0,095 | 0,146 | 0,098 |
| **F8** | 0,137 | 0,241 | 0,089 | 0,232 | 0,787 | 0,601 | 0,824 | 0,527 | 0,446 | 0,300 | 0,286 | 0,406 | 0,442 | 0,625 | 0,278 | 0,699 | 0,135 | 0,161 | 0,181 | 0,274 |
| **T7** | 0,177 | 0,053 | 0,926 | 0,086 | 0,124 | 0,160 | 0,067 | 0,206 | 0,197 | 0,049 | 0,599 | 0,067 | 0,046 | 0,055 | 0,233 | 0,062 | 0,047 | 0,050 | 0,106 | 0,078 |
| **T8** | 0,239 | 0,038 | 0,562 | 0,095 | 0,184 | 0,112 | 0,498 | 0,174 | 0,644 | 0,510 | 0,626 | 0,632 | 0,914 | 0,318 | 0,109 | 0,444 | 0,512 | 0,856 | 0,165 | 0,773 |
| **P7** | 0,839 | 0,971 | 0,256 | 0,919 | 0,358 | 0,253 | 0,542 | 0,433 | 0,418 | 0,226 | 0,437 | 0,352 | 0,341 | 0,233 | 0,205 | 0,396 | 0,118 | 0,086 | 0,076 | 0,227 |
| **P8** | 0,804 | 0,415 | 0,810 | 0,456 | 0,949 | 0,989 | 0,404 | 0,961 | 0,448 | 0,895 | 0,064 | 0,952 | 0,102 | 0,191 | 0,115 | 0,211 | 0,731 | 0,941 | 0,296 | 0,693 |
| **Fz** | 0,070 | 0,027 | 0,492 | 0,037 | 0,002 | 0,001 | 0,061 | 0,001 | 0,548 | 0,269 | 0,801 | 0,313 | 0,672 | 0,587 | 0,991 | 0,551 | 0,248 | 0,348 | 0,252 | 0,466 |
| **Cz** | 0,925 | 0,750 | 0,525 | 0,911 | 0,728 | 0,576 | 0,855 | 0,704 | 0,181 | 0,075 | 0,303 | 0,118 | 0,984 | 0,678 | 0,647 | 0,769 | 0,829 | 0,650 | 0,325 | 0,640 |
| **Pz** | 0,249 | 0,030 | 0,874 | 0,066 | 0,194 | 0,289 | 0,098 | 0,260 | 0,373 | 0,138 | 0,833 | 0,137 | 0,751 | 0,671 | 0,771 | 0,736 | 0,293 | 0,396 | 0,328 | 0,680 |
| **Iz** | 0,653 | 0,185 | 0,674 | 0,268 | 0,845 | 0,867 | 0,422 | 0,931 | 0,449 | 0,268 | 0,666 | 0,290 | 0,647 | 0,456 | 0,649 | 0,554 | 0,051 | 0,047 | 0,062 | 0,109 |
| **FC1** | 0,592 | 0,980 | 0,266 | 0,920 | 0,663 | 0,773 | 0,413 | 0,745 | 0,213 | 0,395 | 0,039 | 0,406 | 0,957 | 0,379 | 0,389 | 0,399 | 0,930 | 0,509 | 0,499 | 0,416 |
| **FC2** | 0,897 | 0,466 | 0,371 | 0,490 | 0,055 | 0,045 | 0,022 | 0,042 | 0,940 | 0,707 | 0,573 | 0,610 | 0,914 | 0,612 | 0,886 | 0,584 | 0,899 | 0,611 | 0,591 | 0,499 |
| **CP1** | 0,198 | 0,032 | 0,954 | 0,053 | 0,033 | 0,078 | 0,014 | 0,073 | 0,365 | 0,092 | 0,977 | 0,131 | 0,345 | 0,241 | 0,631 | 0,283 | 0,693 | 0,903 | 0,449 | 0,651 |
| **CP2** | 0,522 | 0,149 | 0,737 | 0,228 | 0,070 | 0,169 | 0,019 | 0,142 | 0,414 | 0,105 | 0,565 | 0,121 | 0,878 | 0,842 | 0,636 | 0,971 | 0,660 | 0,982 | 0,516 | 0,759 |
| **FC5** | 0,701 | 0,237 | 0,392 | 0,424 | 0,120 | 0,019 | 0,747 | 0,064 | 0,954 | 0,941 | 0,780 | 0,882 | 0,667 | 0,353 | 0,690 | 0,503 | 0,449 | 0,101 | 0,425 | 0,166 |
| **FC6** | 0,341 | 0,854 | 0,035 | 0,551 | 0,089 | 0,094 | 0,255 | 0,097 | 0,974 | 0,619 | 0,923 | 0,688 | 0,993 | 0,571 | 0,356 | 0,734 | 0,745 | 0,976 | 0,253 | 0,935 |
| **CP5** | 0,734 | 0,721 | 0,400 | 0,907 | 0,907 | 0,755 | 0,934 | 0,906 | 0,478 | 0,222 | 0,650 | 0,353 | 0,654 | 0,561 | 0,392 | 0,705 | 0,084 | 0,088 | 0,049 | 0,195 |
| **CP6** | 0,457 | 0,610 | 0,817 | 0,664 | 0,566 | 0,883 | 0,127 | 0,779 | 0,591 | 0,687 | 0,072 | 0,752 | 0,588 | 0,714 | 0,709 | 0,858 | 0,331 | 0,377 | 0,196 | 0,376 |
| **TP9** | 0,175 | 0,019 | 0,642 | 0,058 | 0,272 | 0,127 | 0,624 | 0,185 | 0,345 | 0,216 | 0,416 | 0,222 | 0,997 | 0,900 | 0,708 | 0,988 | 0,096 | 0,149 | 0,097 | 0,272 |
| **TP10** | 0,249 | 0,054 | 0,646 | 0,134 | 0,169 | 0,043 | 0,970 | 0,101 | 0,583 | 0,405 | 0,593 | 0,502 | 0,746 | 0,452 | 0,498 | 0,682 | 0,662 | 0,924 | 0,103 | 0,991 |
| **FT9** | 0,059 | 0,003 | 0,968 | 0,007 | 0,380 | 0,265 | 0,645 | 0,297 | 0,314 | 0,173 | 0,370 | 0,239 | 0,924 | 0,990 | 0,462 | 0,997 | 0,169 | 0,275 | 0,124 | 0,409 |
| **FT10** | 0,012 | 0,004 | 0,266 | 0,004 | 0,428 | 0,554 | 0,176 | 0,579 | 0,489 | 0,250 | 0,618 | 0,290 | 0,919 | 0,819 | 0,595 | 0,789 | 0,156 | 0,153 | 0,086 | 0,227 |
| **F1** | 0,191 | 0,097 | 0,173 | 0,221 | 0,280 | 0,165 | 0,278 | 0,357 | 0,250 | 0,114 | 0,241 | 0,261 | 0,147 | 0,077 | 0,154 | 0,164 | 0,089 | 0,056 | 0,071 | 0,115 |
| **F2** | 0,283 | 0,291 | 0,213 | 0,497 | 0,118 | 0,253 | 0,242 | 0,115 | 0,338 | 0,137 | 0,392 | 0,277 | 0,266 | 0,209 | 0,196 | 0,336 | 0,105 | 0,081 | 0,123 | 0,170 |
| **C1** | 0,425 | 0,127 | 0,923 | 0,104 | 0,011 | 0,009 | 0,023 | 0,010 | 0,483 | 0,194 | 0,772 | 0,256 | 0,060 | 0,014 | 0,551 | 0,014 | 0,683 | 0,297 | 0,877 | 0,196 |
| **C2** | 0,783 | 0,482 | 0,886 | 0,510 | 0,314 | 0,659 | 0,133 | 0,462 | 0,372 | 0,120 | 0,996 | 0,163 | 0,884 | 0,821 | 0,846 | 0,879 | 0,771 | 0,848 | 0,462 | 0,636 |
| **P1** | 0,371 | 0,126 | 0,897 | 0,144 | 0,389 | 0,626 | 0,152 | 0,500 | 0,252 | 0,057 | 0,966 | 0,084 | 0,931 | 0,906 | 0,996 | 0,854 | 0,441 | 0,646 | 0,475 | 0,935 |
| **P2** | 0,504 | 0,137 | 0,812 | 0,218 | 0,304 | 0,440 | 0,104 | 0,384 | 0,516 | 0,153 | 0,453 | 0,144 | 0,868 | 0,939 | 0,592 | 0,972 | 0,656 | 0,982 | 0,537 | 0,763 |
| **AF3** | 0,936 | 0,369 | 0,281 | 0,489 | 0,094 | 0,039 | 0,584 | 0,036 | 0,179 | 0,100 | 0,141 | 0,148 | 0,158 | 0,187 | 0,187 | 0,183 | 0,150 | 0,179 | 0,171 | 0,190 |
| **AF4** | 0,259 | 0,488 | 0,118 | 0,487 | 0,167 | 0,146 | 0,409 | 0,152 | 0,167 | 0,052 | 0,231 | 0,104 | 0,205 | 0,184 | 0,217 | 0,251 | 0,152 | 0,126 | 0,189 | 0,190 |
| **FC3** | 0,609 | 0,107 | 0,431 | 0,214 | 0,058 | 0,006 | 0,342 | 0,013 | 0,587 | 0,726 | 0,872 | 0,848 | 0,337 | 0,088 | 0,709 | 0,201 | 0,402 | 0,059 | 0,435 | 0,102 |
| **FC4** | 0,841 | 0,532 | 0,170 | 0,636 | 0,033 | 0,064 | 0,046 | 0,044 | 0,973 | 0,625 | 0,868 | 0,685 | 0,639 | 0,346 | 0,442 | 0,395 | 0,608 | 0,897 | 0,220 | 0,987 |
| **CP3** | 0,204 | 0,048 | 0,529 | 0,067 | 0,181 | 0,351 | 0,044 | 0,315 | 0,750 | 0,219 | 0,392 | 0,277 | 0,314 | 0,205 | 0,669 | 0,247 | 0,739 | 0,796 | 0,483 | 0,614 |
| **CP4** | 0,218 | 0,024 | 0,841 | 0,056 | 0,167 | 0,403 | 0,025 | 0,329 | 0,717 | 0,533 | 0,074 | 0,611 | 0,339 | 0,418 | 0,395 | 0,508 | 0,869 | 0,801 | 0,533 | 0,601 |
| **PO3** | 0,950 | 0,830 | 0,920 | 0,796 | 0,943 | 0,844 | 0,482 | 0,953 | 0,350 | 0,148 | 0,820 | 0,163 | 0,517 | 0,522 | 0,842 | 0,596 | 0,071 | 0,091 | 0,212 | 0,211 |
| **PO4** | 0,655 | 0,760 | 0,840 | 0,805 | 0,899 | 0,954 | 0,244 | 0,988 | 0,896 | 0,417 | 0,354 | 0,354 | 0,882 | 0,694 | 0,234 | 0,671 | 0,099 | 0,108 | 0,199 | 0,212 |
| **F5** | 0,737 | 0,663 | 0,157 | 0,932 | 0,371 | 0,094 | 0,772 | 0,155 | 0,996 | 0,814 | 0,335 | 0,909 | 0,794 | 0,254 | 0,268 | 0,381 | 0,683 | 0,184 | 0,335 | 0,313 |
| **F6** | 0,078 | 0,190 | 0,060 | 0,153 | 0,311 | 0,306 | 0,407 | 0,251 | 0,380 | 0,183 | 0,443 | 0,314 | 0,679 | 0,980 | 0,205 | 0,947 | 0,383 | 0,582 | 0,102 | 0,750 |
| **C5** | 0,341 | 0,048 | 0,461 | 0,131 | 0,107 | 0,061 | 0,229 | 0,112 | 0,696 | 0,323 | 0,840 | 0,356 | 0,742 | 0,974 | 0,425 | 0,823 | 0,128 | 0,169 | 0,191 | 0,165 |
| **C6** | 0,279 | 0,047 | 0,580 | 0,115 | 0,092 | 0,131 | 0,206 | 0,116 | 0,937 | 0,605 | 0,981 | 0,705 | 0,212 | 0,319 | 0,053 | 0,303 | 0,084 | 0,136 | 0,037 | 0,144 |
| **P5** | 0,287 | 0,234 | 0,453 | 0,445 | 0,491 | 0,319 | 0,924 | 0,519 | 0,328 | 0,108 | 0,729 | 0,196 | 0,222 | 0,156 | 0,285 | 0,317 | 0,059 | 0,076 | 0,108 | 0,250 |
| **P6** | 0,499 | 0,542 | 0,998 | 0,616 | 1,000 | 0,774 | 0,241 | 0,828 | 0,477 | 0,767 | 0,042 | 0,749 | 0,421 | 0,785 | 0,126 | 0,930 | 0,089 | 0,092 | 0,199 | 0,201 |
| **AF7** | 0,703 | 0,949 | 0,199 | 0,860 | 0,486 | 0,336 | 0,737 | 0,417 | 0,220 | 0,208 | 0,119 | 0,218 | 0,656 | 0,845 | 0,268 | 0,971 | 0,375 | 0,756 | 0,147 | 0,747 |
| **AF8** | 0,642 | 0,931 | 0,299 | 0,789 | 0,393 | 0,199 | 0,961 | 0,241 | 0,267 | 0,180 | 0,160 | 0,257 | 0,389 | 0,495 | 0,278 | 0,694 | 0,176 | 0,174 | 0,173 | 0,280 |
| **FT7** | 0,285 | 0,023 | 0,426 | 0,074 | 0,192 | 0,029 | 0,901 | 0,080 | 0,596 | 0,502 | 0,546 | 0,474 | 0,532 | 0,878 | 0,216 | 0,694 | 0,210 | 0,366 | 0,131 | 0,378 |
| **FT8** | 0,417 | 0,097 | 0,355 | 0,194 | 0,123 | 0,065 | 0,587 | 0,089 | 0,916 | 0,930 | 0,499 | 0,907 | 0,701 | 0,251 | 0,295 | 0,310 | 0,668 | 0,961 | 0,246 | 0,959 |
| **TP7** | 0,336 | 0,234 | 0,232 | 0,459 | 0,277 | 0,149 | 0,354 | 0,335 | 0,292 | 0,128 | 0,326 | 0,267 | 0,130 | 0,053 | 0,181 | 0,142 | 0,043 | 0,023 | 0,049 | 0,087 |
| **TP8** | 0,291 | 0,052 | 0,612 | 0,117 | 0,642 | 0,603 | 0,491 | 0,657 | 0,955 | 0,741 | 0,995 | 0,723 | 0,858 | 0,590 | 0,517 | 0,759 | 0,379 | 0,835 | 0,049 | 0,926 |
| **PO7** | 0,875 | 0,753 | 0,944 | 0,788 | 0,586 | 0,516 | 0,742 | 0,609 | 0,367 | 0,198 | 0,952 | 0,226 | 0,259 | 0,283 | 0,445 | 0,286 | 0,052 | 0,072 | 0,099 | 0,129 |
| **PO8** | 0,556 | 0,644 | 0,949 | 0,711 | 0,558 | 0,430 | 0,489 | 0,490 | 0,805 | 0,657 | 0,213 | 0,648 | 0,568 | 0,857 | 0,279 | 0,847 | 0,091 | 0,128 | 0,098 | 0,186 |
| **Fpz** | 0,000 | 0,002 | 0,001 | 0,002 | 0,001 | 0,003 | 0,037 | 0,005 | 0,464 | 0,184 | 0,506 | 0,248 | 0,541 | 0,437 | 0,624 | 0,409 | 0,200 | 0,173 | 0,252 | 0,190 |
| **CPz** | 0,365 | 0,290 | 0,223 | 0,476 | 0,723 | 0,393 | 0,929 | 0,660 | 0,163 | 0,039 | 0,532 | 0,078 | 0,300 | 0,168 | 0,348 | 0,350 | 0,262 | 0,183 | 0,248 | 0,423 |
| **Poz** | 0,734 | 0,916 | 0,884 | 0,952 | 0,872 | 0,705 | 0,526 | 0,848 | 0,324 | 0,122 | 0,972 | 0,114 | 0,721 | 0,501 | 1,000 | 0,640 | 0,169 | 0,238 | 0,270 | 0,395 |
| **Oz** | 0,880 | 0,468 | 0,899 | 0,582 | 0,857 | 0,825 | 0,607 | 0,837 | 0,395 | 0,217 | 0,763 | 0,217 | 0,608 | 0,501 | 0,752 | 0,538 | 0,087 | 0,110 | 0,162 | 0,159 |

**Table 1:** Correlation between heart-rate variability (HRV) and cortical oscillatory activity (EEG): P-values of cymba conchae/inner tragus stimulation
