# Supplementary material for: Brain–Heart Interaction During Transcutaneous Auricular Vagus Nerve Stimulation
Source: Front Neurosci. 2021 Mar 15;15:632697. doi: 10.3389/fnins.2021.632697 (PMC8005577; doi:10.3389/fnins.2021.632697)
Supplement: Supplementary file 2 [file Table_2.docx]

|  | | **DELTA** | | | | **THETA** | | | | **ALPHA** | | | | **BETA** | | | | **GAMMA** | | | |
| --- | --- | --- | --- | --- | --- | --- | --- | --- | --- | --- | --- | --- | --- | --- | --- | --- | --- | --- | --- | --- | --- |
|  | **RMSSD** | | **pNN50** | **SDNN** | **rrHRV** | **RMSSD** | **pNN50** | **SDNN** | **rrHRV** | **RMSSD** | **pNN50** | **SDNN** | **rrHRV** | **RMSSD** | **pNN50** | **SDNN** | **rrHRV** | **RMSSD** | **pNN50** | **SDNN** | **rrHRV** |
| **Fp1** | 0,191 | | 0,279 | 0,414 | 0,370 | 0,000 | 0,000 | 0,062 | 0,000 | 0,811 | 0,577 | 0,145 | 0,449 | 0,956 | 0,989 | 0,000 | 0,819 | 0,946 | 0,954 | 0,000 | 0,780 |
| **Fp2** | 0,174 | | 0,228 | 0,573 | 0,296 | 0,000 | 0,000 | 0,144 | 0,000 | 0,922 | 0,633 | 0,942 | 0,559 | 0,018 | 0,009 | 0,016 | 0,011 | 0,252 | 0,148 | 0,001 | 0,122 |
| **F3** | 0,463 | | 0,459 | 0,512 | 0,228 | 0,002 | 0,001 | 0,986 | 0,000 | 0,506 | 0,535 | 0,297 | 0,343 | 0,076 | 0,023 | 0,665 | 0,009 | 0,026 | 0,005 | 0,684 | 0,001 |
| **F4** | 0,675 | | 0,653 | 0,925 | 0,777 | 0,006 | 0,006 | 0,085 | 0,001 | 0,651 | 0,366 | 0,650 | 0,585 | 0,950 | 0,966 | 0,000 | 0,831 | 0,678 | 0,632 | 0,000 | 0,793 |
| **C3** | 0,222 | | 0,228 | 0,941 | 0,212 | 0,008 | 0,015 | 0,185 | 0,014 | 0,820 | 0,601 | 0,648 | 0,624 | 0,160 | 0,276 | 0,276 | 0,299 | 0,614 | 0,581 | 0,018 | 0,394 |
| **C4** | 0,805 | | 0,553 | 0,542 | 0,647 | 0,084 | 0,102 | 0,344 | 0,165 | 0,800 | 0,577 | 0,891 | 0,656 | 0,199 | 0,341 | 0,091 | 0,356 | 0,891 | 0,815 | 0,006 | 0,876 |
| **P3** | 0,862 | | 0,797 | 0,620 | 0,788 | 0,468 | 0,639 | 0,455 | 0,343 | 0,242 | 0,184 | 0,826 | 0,322 | 0,872 | 0,629 | 0,630 | 0,759 | 0,276 | 0,270 | 0,024 | 0,467 |
| **P4** | 0,837 | | 0,897 | 0,914 | 0,808 | 0,294 | 0,362 | 0,683 | 0,264 | 0,232 | 0,171 | 0,732 | 0,227 | 0,884 | 0,591 | 0,607 | 0,658 | 0,382 | 0,313 | 0,018 | 0,654 |
| **O1** | 0,877 | | 0,894 | 0,620 | 0,718 | 0,469 | 0,624 | 0,396 | 0,380 | 0,327 | 0,301 | 0,761 | 0,391 | 0,456 | 0,300 | 0,290 | 0,455 | 0,243 | 0,184 | 0,032 | 0,169 |
| **O2** | 0,607 | | 0,578 | 0,728 | 0,882 | 0,447 | 0,602 | 0,562 | 0,358 | 0,370 | 0,342 | 0,745 | 0,365 | 0,934 | 0,714 | 0,224 | 0,869 | 0,258 | 0,240 | 0,045 | 0,685 |
| **F7** | 0,200 | | 0,186 | 0,137 | 0,238 | 0,003 | 0,003 | 0,086 | 0,003 | 0,672 | 0,523 | 0,054 | 0,746 | 0,770 | 0,695 | 0,001 | 0,739 | 0,625 | 0,565 | 0,001 | 0,675 |
| **F8** | 0,802 | | 0,765 | 0,756 | 0,679 | 0,027 | 0,050 | 0,234 | 0,029 | 0,673 | 0,348 | 0,769 | 0,430 | 0,550 | 0,508 | 0,000 | 0,216 | 0,679 | 0,744 | 0,010 | 0,812 |
| **T7** | 0,529 | | 0,574 | 0,584 | 0,512 | 0,015 | 0,021 | 0,111 | 0,016 | 0,436 | 0,286 | 0,016 | 0,482 | 0,716 | 0,663 | 0,001 | 0,803 | 0,446 | 0,388 | 0,003 | 0,486 |
| **T8** | 0,901 | | 0,800 | 0,640 | 0,676 | 0,032 | 0,053 | 0,419 | 0,039 | 0,732 | 0,408 | 0,744 | 0,559 | 0,977 | 0,991 | 0,008 | 0,609 | 0,414 | 0,354 | 0,060 | 0,721 |
| **P7** | 0,760 | | 0,614 | 0,736 | 0,947 | 0,930 | 0,721 | 0,481 | 0,894 | 0,507 | 0,357 | 0,786 | 0,628 | 0,598 | 0,485 | 0,168 | 0,682 | 0,470 | 0,463 | 0,156 | 0,654 |
| **P8** | 0,473 | | 0,444 | 0,925 | 0,734 | 0,724 | 0,618 | 0,842 | 0,907 | 0,449 | 0,298 | 0,786 | 0,505 | 0,691 | 0,520 | 0,597 | 0,741 | 0,369 | 0,393 | 0,564 | 0,608 |
| **Fz** | 0,000 | | 0,000 | 0,210 | 0,000 | 0,000 | 0,000 | 0,150 | 0,000 | 0,632 | 0,559 | 0,699 | 0,493 | 0,112 | 0,189 | 0,005 | 0,220 | 0,995 | 0,920 | 0,000 | 0,733 |
| **Cz** | 0,882 | | 0,963 | 0,515 | 0,899 | 0,075 | 0,046 | 0,995 | 0,046 | 0,174 | 0,121 | 0,316 | 0,176 | 0,638 | 0,650 | 0,638 | 0,560 | 0,886 | 0,515 | 0,689 | 0,346 |
| **Pz** | 0,112 | | 0,054 | 0,358 | 0,042 | 0,013 | 0,020 | 0,270 | 0,011 | 0,328 | 0,308 | 0,519 | 0,358 | 0,919 | 0,885 | 0,654 | 0,868 | 0,647 | 0,793 | 0,005 | 0,948 |
| **Iz** | 0,640 | | 0,668 | 0,928 | 0,964 | 0,136 | 0,203 | 0,371 | 0,103 | 0,477 | 0,495 | 0,526 | 0,414 | 0,810 | 0,470 | 0,680 | 0,745 | 0,136 | 0,105 | 0,136 | 0,310 |
| **FC1** | 0,166 | | 0,225 | 0,073 | 0,460 | 0,846 | 0,796 | 0,341 | 0,410 | 0,553 | 0,525 | 0,812 | 0,728 | 0,867 | 0,841 | 0,671 | 0,604 | 0,838 | 0,490 | 0,728 | 0,368 |
| **FC2** | 0,283 | | 0,254 | 0,066 | 0,728 | 0,989 | 1,000 | 0,760 | 0,591 | 0,790 | 0,536 | 0,909 | 0,751 | 0,600 | 0,641 | 0,979 | 0,486 | 0,854 | 0,543 | 0,710 | 0,352 |
| **CP1** | 0,294 | | 0,173 | 0,847 | 0,147 | 0,034 | 0,051 | 0,355 | 0,035 | 0,226 | 0,160 | 0,574 | 0,217 | 0,688 | 0,779 | 0,861 | 0,874 | 0,736 | 0,707 | 0,247 | 0,526 |
| **CP2** | 0,421 | | 0,295 | 0,706 | 0,567 | 0,018 | 0,025 | 0,457 | 0,026 | 0,209 | 0,141 | 0,390 | 0,196 | 0,963 | 0,865 | 0,746 | 0,823 | 0,817 | 0,757 | 0,155 | 0,525 |
| **FC5** | 0,049 | | 0,032 | 0,647 | 0,045 | 0,000 | 0,000 | 0,194 | 0,001 | 0,685 | 0,819 | 0,037 | 0,938 | 0,100 | 0,082 | 0,065 | 0,100 | 0,228 | 0,098 | 0,011 | 0,124 |
| **FC6** | 0,844 | | 0,880 | 0,956 | 0,888 | 0,014 | 0,014 | 0,234 | 0,013 | 0,855 | 0,767 | 0,516 | 0,915 | 0,411 | 0,498 | 0,005 | 0,263 | 0,736 | 0,775 | 0,097 | 0,761 |
| **CP5** | 0,507 | | 0,448 | 0,534 | 0,250 | 0,309 | 0,413 | 0,325 | 0,208 | 0,529 | 0,384 | 0,499 | 0,611 | 0,590 | 0,793 | 0,165 | 0,671 | 0,348 | 0,475 | 0,160 | 0,476 |
| **CP6** | 0,579 | | 0,623 | 0,853 | 0,893 | 0,366 | 0,453 | 0,677 | 0,349 | 0,447 | 0,308 | 0,832 | 0,437 | 0,568 | 0,844 | 0,066 | 0,734 | 0,631 | 0,642 | 0,038 | 0,917 |
| **TP9** | 0,027 | | 0,029 | 0,425 | 0,022 | 0,003 | 0,004 | 0,120 | 0,004 | 0,915 | 0,854 | 0,170 | 0,889 | 0,817 | 0,913 | 0,046 | 0,673 | 0,568 | 0,633 | 0,111 | 0,885 |
| **TP10** | 0,599 | | 0,485 | 0,986 | 0,769 | 0,020 | 0,012 | 0,441 | 0,020 | 0,960 | 0,743 | 0,895 | 0,675 | 0,368 | 0,468 | 0,045 | 0,242 | 0,330 | 0,357 | 0,066 | 0,399 |
| **FT9** | 0,000 | | 0,000 | 0,026 | 0,000 | 0,015 | 0,044 | 0,151 | 0,024 | 0,586 | 0,898 | 0,277 | 0,741 | 0,151 | 0,303 | 0,000 | 0,317 | 0,728 | 0,854 | 0,000 | 0,844 |
| **FT10** | 0,998 | | 0,878 | 0,264 | 0,601 | 0,487 | 0,546 | 0,405 | 0,362 | 0,298 | 0,243 | 0,802 | 0,297 | 0,776 | 0,797 | 0,000 | 0,380 | 0,614 | 0,659 | 0,003 | 0,967 |
| **F1** | 0,401 | | 0,281 | 0,301 | 0,296 | 0,347 | 0,400 | 0,696 | 0,480 | 0,405 | 0,320 | 0,320 | 0,309 | 0,624 | 0,593 | 0,573 | 0,553 | 0,422 | 0,437 | 0,782 | 0,419 |
| **F2** | 0,757 | | 0,721 | 0,715 | 0,933 | 0,001 | 0,000 | 0,142 | 0,001 | 0,509 | 0,395 | 0,754 | 0,447 | 0,831 | 0,946 | 0,008 | 0,819 | 0,643 | 0,622 | 0,000 | 0,845 |
| **C1** | 0,046 | | 0,005 | 0,893 | 0,003 | 0,007 | 0,009 | 0,381 | 0,012 | 0,245 | 0,158 | 0,488 | 0,188 | 0,174 | 0,187 | 0,610 | 0,224 | 0,546 | 0,134 | 0,394 | 0,093 |
| **C2** | 0,791 | | 0,926 | 0,105 | 0,777 | 0,058 | 0,082 | 0,754 | 0,066 | 0,229 | 0,144 | 0,264 | 0,178 | 0,753 | 0,861 | 0,954 | 0,972 | 0,941 | 0,603 | 0,559 | 0,429 |
| **P1** | 0,232 | | 0,153 | 0,190 | 0,116 | 0,076 | 0,138 | 0,328 | 0,073 | 0,217 | 0,179 | 0,623 | 0,261 | 0,928 | 0,872 | 0,647 | 0,894 | 0,582 | 0,790 | 0,003 | 0,965 |
| **P2** | 0,204 | | 0,096 | 0,516 | 0,069 | 0,043 | 0,062 | 0,440 | 0,041 | 0,237 | 0,200 | 0,517 | 0,244 | 0,923 | 0,859 | 0,590 | 0,903 | 0,834 | 0,742 | 0,022 | 0,393 |
| **AF3** | 0,289 | | 0,224 | 0,104 | 0,245 | 0,000 | 0,000 | 0,083 | 0,000 | 0,491 | 0,404 | 0,916 | 0,306 | 0,279 | 0,255 | 0,002 | 0,373 | 0,842 | 0,784 | 0,001 | 0,895 |
| **AF4** | 0,762 | | 0,760 | 0,892 | 0,848 | 0,002 | 0,001 | 0,036 | 0,001 | 0,389 | 0,236 | 0,999 | 0,259 | 0,995 | 0,926 | 0,001 | 0,982 | 0,739 | 0,708 | 0,000 | 0,857 |
| **FC3** | 0,026 | | 0,019 | 0,509 | 0,007 | 0,000 | 0,000 | 0,152 | 0,000 | 0,157 | 0,226 | 0,070 | 0,400 | 0,081 | 0,053 | 0,123 | 0,100 | 0,441 | 0,126 | 0,149 | 0,148 |
| **FC4** | 0,917 | | 0,828 | 0,895 | 0,406 | 0,049 | 0,041 | 0,124 | 0,022 | 0,821 | 0,910 | 0,524 | 0,990 | 0,218 | 0,269 | 0,123 | 0,195 | 0,867 | 0,919 | 0,201 | 0,955 |
| **CP3** | 0,384 | | 0,356 | 0,436 | 0,193 | 0,191 | 0,297 | 0,302 | 0,169 | 0,395 | 0,272 | 0,822 | 0,409 | 0,387 | 0,483 | 0,413 | 0,450 | 0,738 | 0,715 | 0,173 | 0,556 |
| **CP4** | 0,440 | | 0,259 | 0,947 | 0,167 | 0,064 | 0,092 | 0,518 | 0,100 | 0,396 | 0,270 | 0,910 | 0,382 | 0,451 | 0,632 | 0,289 | 0,637 | 0,968 | 0,671 | 0,008 | 0,346 |
| **PO3** | 0,984 | | 0,975 | 0,534 | 0,580 | 0,674 | 0,873 | 0,491 | 0,517 | 0,281 | 0,247 | 0,806 | 0,393 | 0,551 | 0,412 | 0,457 | 0,671 | 0,265 | 0,313 | 0,041 | 0,625 |
| **PO4** | 0,805 | | 0,841 | 0,767 | 0,896 | 0,425 | 0,585 | 0,584 | 0,395 | 0,298 | 0,271 | 0,722 | 0,296 | 0,925 | 0,630 | 0,623 | 0,842 | 0,320 | 0,374 | 0,182 | 0,796 |
| **F5** | 0,749 | | 0,744 | 0,392 | 0,819 | 0,001 | 0,000 | 0,101 | 0,001 | 0,693 | 0,747 | 0,092 | 0,754 | 0,051 | 0,018 | 0,113 | 0,031 | 0,058 | 0,048 | 0,180 | 0,145 |
| **F6** | 0,882 | | 0,837 | 0,441 | 0,863 | 0,017 | 0,017 | 0,073 | 0,010 | 0,967 | 0,741 | 0,919 | 0,787 | 0,422 | 0,271 | 0,042 | 0,171 | 0,483 | 0,251 | 0,049 | 0,187 |
| **C5** | 0,061 | | 0,027 | 0,498 | 0,016 | 0,004 | 0,007 | 0,137 | 0,005 | 0,852 | 0,627 | 0,040 | 0,748 | 0,700 | 0,830 | 0,001 | 0,819 | 0,856 | 0,865 | 0,001 | 0,770 |
| **C6** | 0,907 | | 0,949 | 0,874 | 0,993 | 0,080 | 0,153 | 0,540 | 0,162 | 0,643 | 0,368 | 0,534 | 0,477 | 0,979 | 0,851 | 0,005 | 0,886 | 0,643 | 0,578 | 0,015 | 0,561 |
| **P5** | 0,459 | | 0,382 | 0,916 | 0,687 | 0,977 | 0,778 | 0,506 | 0,783 | 0,346 | 0,267 | 0,991 | 0,472 | 0,884 | 0,706 | 0,312 | 0,983 | 0,257 | 0,371 | 0,088 | 0,650 |
| **P6** | 0,555 | | 0,545 | 0,947 | 0,836 | 0,898 | 0,941 | 0,970 | 0,896 | 0,315 | 0,243 | 0,870 | 0,299 | 0,738 | 0,503 | 0,784 | 0,567 | 0,677 | 0,715 | 0,057 | 0,872 |
| **AF7** | 0,963 | | 0,941 | 0,643 | 0,934 | 0,001 | 0,001 | 0,110 | 0,001 | 0,912 | 0,762 | 0,312 | 0,759 | 0,322 | 0,164 | 0,133 | 0,176 | 0,503 | 0,402 | 0,011 | 0,392 |
| **AF8** | 0,832 | | 0,908 | 0,499 | 0,909 | 0,003 | 0,007 | 0,050 | 0,006 | 0,579 | 0,366 | 0,526 | 0,439 | 0,680 | 0,418 | 0,004 | 0,236 | 0,821 | 0,937 | 0,001 | 0,849 |
| **FT7** | 0,181 | | 0,205 | 0,530 | 0,204 | 0,007 | 0,010 | 0,148 | 0,008 | 0,855 | 0,638 | 0,075 | 0,785 | 0,936 | 0,910 | 0,000 | 0,832 | 0,637 | 0,606 | 0,003 | 0,714 |
| **FT8** | 0,966 | | 0,962 | 0,960 | 0,952 | 0,018 | 0,030 | 0,340 | 0,015 | 0,608 | 0,347 | 0,861 | 0,453 | 0,504 | 0,253 | 0,271 | 0,101 | 0,526 | 0,844 | 0,504 | 0,903 |
| **TP7** | 0,483 | | 0,498 | 0,579 | 0,373 | 0,237 | 0,400 | 0,318 | 0,242 | 0,832 | 0,543 | 0,390 | 0,732 | 0,898 | 0,904 | 0,001 | 0,593 | 0,647 | 0,642 | 0,001 | 0,497 |
| **TP8** | 0,639 | | 0,502 | 0,621 | 0,308 | 0,420 | 0,480 | 0,544 | 0,325 | 0,479 | 0,348 | 0,741 | 0,455 | 0,676 | 0,913 | 0,009 | 0,796 | 0,559 | 0,550 | 0,006 | 0,726 |
| **PO7** | 0,577 | | 0,519 | 0,664 | 0,946 | 0,744 | 0,593 | 0,512 | 0,998 | 0,321 | 0,257 | 0,960 | 0,491 | 0,444 | 0,321 | 0,310 | 0,649 | 0,507 | 0,456 | 0,029 | 0,820 |
| **PO8** | 0,465 | | 0,433 | 0,788 | 0,748 | 0,917 | 0,764 | 0,738 | 0,924 | 0,379 | 0,308 | 0,919 | 0,405 | 0,617 | 0,915 | 0,236 | 0,458 | 0,982 | 0,850 | 0,094 | 0,571 |
| **Fpz** | 0,039 | | 0,069 | 0,268 | 0,140 | 0,000 | 0,000 | 0,082 | 0,002 | 0,447 | 0,290 | 0,610 | 0,172 | 0,880 | 0,923 | 0,000 | 0,632 | 0,843 | 0,805 | 0,000 | 0,933 |
| **CPz** | 0,401 | | 0,327 | 0,252 | 0,341 | 0,963 | 0,802 | 0,421 | 0,903 | 0,209 | 0,152 | 0,225 | 0,178 | 0,432 | 0,415 | 0,406 | 0,388 | 0,447 | 0,487 | 0,483 | 0,493 |
| **Poz** | 0,297 | | 0,167 | 0,250 | 0,130 | 0,035 | 0,076 | 0,197 | 0,044 | 0,394 | 0,407 | 0,641 | 0,402 | 0,737 | 0,957 | 0,307 | 0,877 | 0,727 | 0,908 | 0,002 | 0,777 |
| **Oz** | 0,884 | | 0,944 | 0,627 | 0,762 | 0,140 | 0,215 | 0,377 | 0,112 | 0,314 | 0,292 | 0,643 | 0,337 | 0,984 | 0,700 | 0,260 | 0,997 | 0,312 | 0,306 | 0,005 | 0,598 |

**Table 2:** Correlation between heart-rate variability (HRV) and cortical oscillatory activity (EEG): P-values of outer tragus/crus helicis stimulation
